# Safety and tolerability of intranasal insulin in Parkinson’s disease

**DOI:** 10.3389/fneur.2026.1875165

**Published:** 2026-09-09

**Authors:** Julia C. Johnson, Bhavani Kashyap, Sally K. Gustafson, Meghan O'Brien, William Howard Frey, Aleta L. Svitak, Leah R. Hanson

**Affiliations:** 1 Struthers Parkinson’s Center at HealthPartners, St Paul, MN, United States; 2 HealthPartners Institute, Bloomington, MN, United States

**Keywords:** clinical trial, cognitive impairment, glucose, nose to brain delivery, phase II

## Abstract

**Background:**

Repurposing therapeutic treatments for diabetes may be a promising strategy for the treatment of neurodegenerative disorders. Intranasal insulin (INI) is shown to improve memory, attention, and cognition in Alzheimer’s dementia. Associations between the etiology of Parkinson’s Disease (PD), brain glucose hypometabolism, and central insulin resistance suggest INI as a promising treatment for PD.

**Methods:**

A phase II single center, randomized, double-blind clinical trial was conducted in 30 participants with PD, aged 41–89, MoCA ≥ 16, on a stable dose of anti-PD medications. Participants were randomized to receive intranasal treatment for 21 days with either placebo, or 20, 40, or 80 IU of regular insulin (INI) using SipNose intranasal device. Primary outcomes of safety and tolerability were assessed in 30 participants by monitoring hypoglycemia, weight, and adverse events. Cognitive, mood, and motor measures were collected pre- and post-treatment for secondary outcome analysis and assessed in 28 participants.

**Results:**

No significant changes in weight or serum insulin were observed during the study. No hypoglycemic values (<70 mg/dL) were recorded 15 or 30 min post-IN dose. Nasal irritation and nosebleeds were the most common adverse events, with most categorized as “mild.” Several secondary outcomes (e.g., verbal fluency and recall) improved from baseline in the 40 IU/INI arm only, though not significantly more than in the placebo arm. No other outcomes assessed (mood, apathy, motor) improved under INI treatment.

**Conclusion:**

INI exhibits an acceptable safety profile in patients with PD. Further studies to investigate if INI has a positive impact on cognitive and motor symptoms in PD are warranted.

**Clinical trial registration:**

https://clinicaltrials.gov/study/NCT04251585 Identifier, NCT04251585.

## Introduction

Parkinson’s disease (PD) is the second most prevalent neurodegenerative disorder, following Alzheimer’s disease. In addition to the motor symptoms, PD is recognized as a multisystem disorder, with a wide range of non-motor manifestations, including cognitive impairment, are commonly observed in affected individuals (1). Despite their prevalence, cognitive symptoms in PD remain poorly managed, with limited pharmacological options available.

Recent research has increasingly focused on the role of metabolic dysfunction in PD, particularly insulin signaling and insulin resistance (2). Emerging evidence suggests a significant association between PD and type 2 diabetes mellitus, particularly through shared mechanisms (2) such as mitochondrial dysfunction, inflammation, and impaired glucose metabolism. Notably, authors in studies (3, 4) reported an approximately 38% increased risk of developing PD in patients with baseline diabetes compared to non-diabetic patients. Furthermore, type 2 diabetes mellitus has been linked to accelerated progression of PD, exacerbation of motor and cognitive symptoms, and molecular alterations in the striatum and cerebrospinal fluid (5). A large cohort study by De Pablo-Fernandez et al. reported a higher incidence of PD among individuals with type 2 diabetes mellitus (6), suggesting the possibility of shared genetic predisposition or overlapping pathogenic pathways, although the causal relationships remain incompletely understood.

Mechanistically, insulin receptors are found in the basal ganglia and substantia nigra and there is evidence that insulin signaling plays a role in the regulation of neuronal survival, dopaminergic transmission, synaptic plasticity, and energy metabolism (2, 7). Disruption of this pathway in PD has been linked to altered insulin-like growth factor 1 (IGF-1) levels, insulin receptor substrate (IRS) phosphorylation, and reduced insulin receptor gene expression (8, 9). These changes contribute to impaired cerebral glucose metabolism (10, 11), as evidenced by fluorodeoxyglucose positron emission tomography (FDG-PET) studies showing hypometabolism in multiple cortical regions in PD patients including occipital, temporal, parietal, and frontal regions (12, 13).

Given these insights, there is growing interest in repurposing antidiabetic agents for PD treatment. Several FDA-approved treatments for type 2 diabetes mellitus have shown promise in PD clinical trials, particularly for their effects on mitochondrial function (14). Among these, glucagon-like peptide-1 (GLP-1) receptor agonists such as exenatide have demonstrated beneficial effects on motor and cognitive outcomes in PD patients (15), without evidence to support a disease-modifying effect (16). However, systemic side effects (17) such as weight loss and gastrointestinal symptoms (18) may limit the use of these agents, highlighting the need for alternative delivery strategies.

Intranasal delivery offers a non-invasive route to deliver large molecules such as insulin directly to the brain while minimizing systemic exposure (19, 20). Recent studies with INI using PET scan shows increased brain uptake in multiple brain regions including hippocampus, caudate and thalamus (21, 22). There is also evidence for detection of insulin in the CSF after intranasal delivery (21, 23). These findings provide evidence of central nervous system target engagement and support the investigation of INI as a therapeutic strategy for neurodegenerative disorders, including PD. Clinical investigations of intranasal insulin in healthy adults, Alzheimer’s dementia (AD) have demonstrated therapeutic effects impacting memory, attention, and cognition without significantly altering serum insulin or glucose levels (24–31). Although a longer-duration trial in individuals with mild cognitive impairment and Alzheimer’s disease did not show cognitive or functional benefits (28), INI treatment was associated with reduced progression of white matter hyperintensities in deep and frontal brain regions (32).

To date, only one small randomized, double-blind study has evaluated INI in PD, which reported good tolerability and preliminary improvements in verbal fluency and improvement in motor function measured by UPDRS motor (part III) scores (33). Rodent models of PD have also demonstrated that INI can improve motor function and promote dopaminergic cell survival (34, 35). Moreover, insulin receptors and glucose transporters are co-localized in memory-related brain regions, suggesting that enhanced cerebral glucose utilization may support cognitive function (36, 37).

Previous INI studies have used doses ranging from 10 to 160 IU/INI across various populations. The Novak et al. (2019) study tested only one dose (40 IU) of intranasal insulin (33), which did show preliminary efficacy in select domains. It is unclear whether the chosen dose is optimal for clinical benefit, or whether a higher dose would be safe in this population. In the present study, we evaluated the safety and tolerability of three different doses (20 IU, 40 IU, and 80 IU) of intranasal insulin compared to placebo over a 21-day period in individuals with PD. Additionally, we explored the preliminary effects of INI on cognition, mood, apathy, and motor performance.

## Methods

### Study design

**Figure 1:**
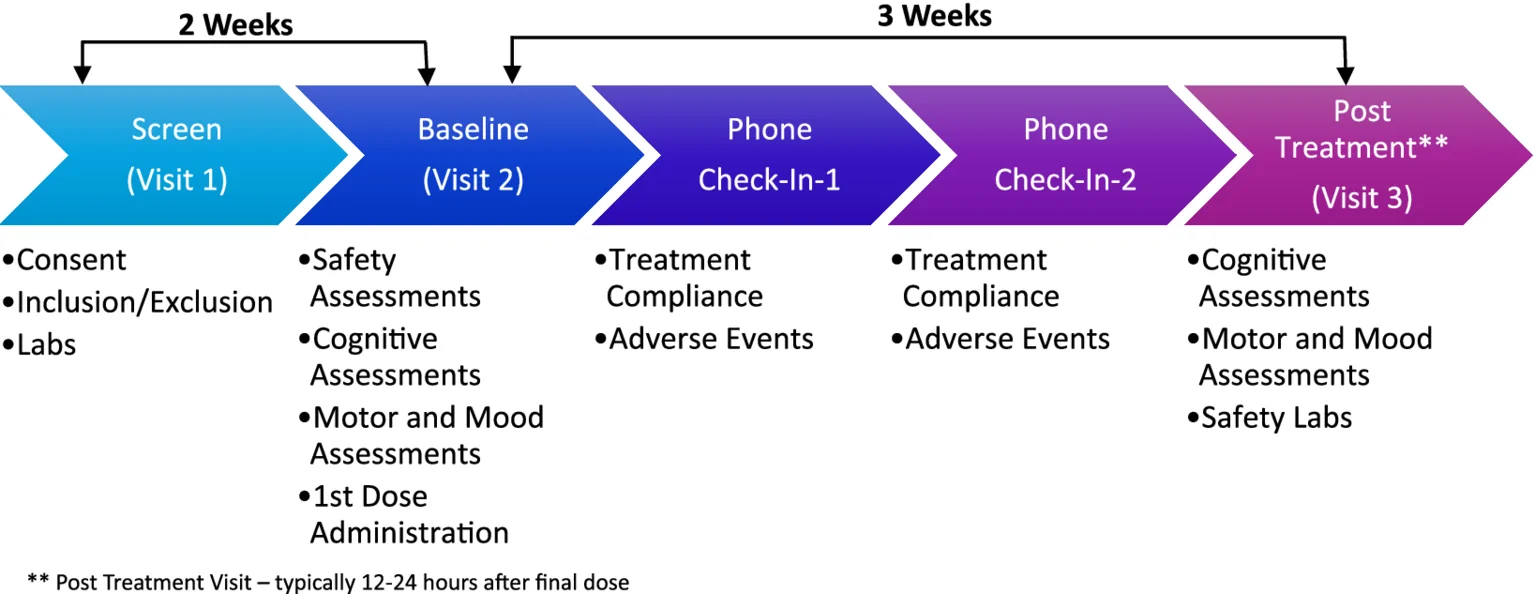
Study design and visits.

This study was a single center, randomized, double-blinded, placebo-controlled, phase 2 trial to determine dose finding, safety and tolerability of three doses of intranasal (IN) regular insulin (Novolin R) compared to placebo (saline) in patients with PD (Figure 1). The study was conducted at HealthPartners Neuroscience Center in St. Paul, Minnesota. The study was approved by local institutional review board (IRB) and registered on www.clinicaltrials.gov (NCT04251585).

### Study participants

**Figure 2:**
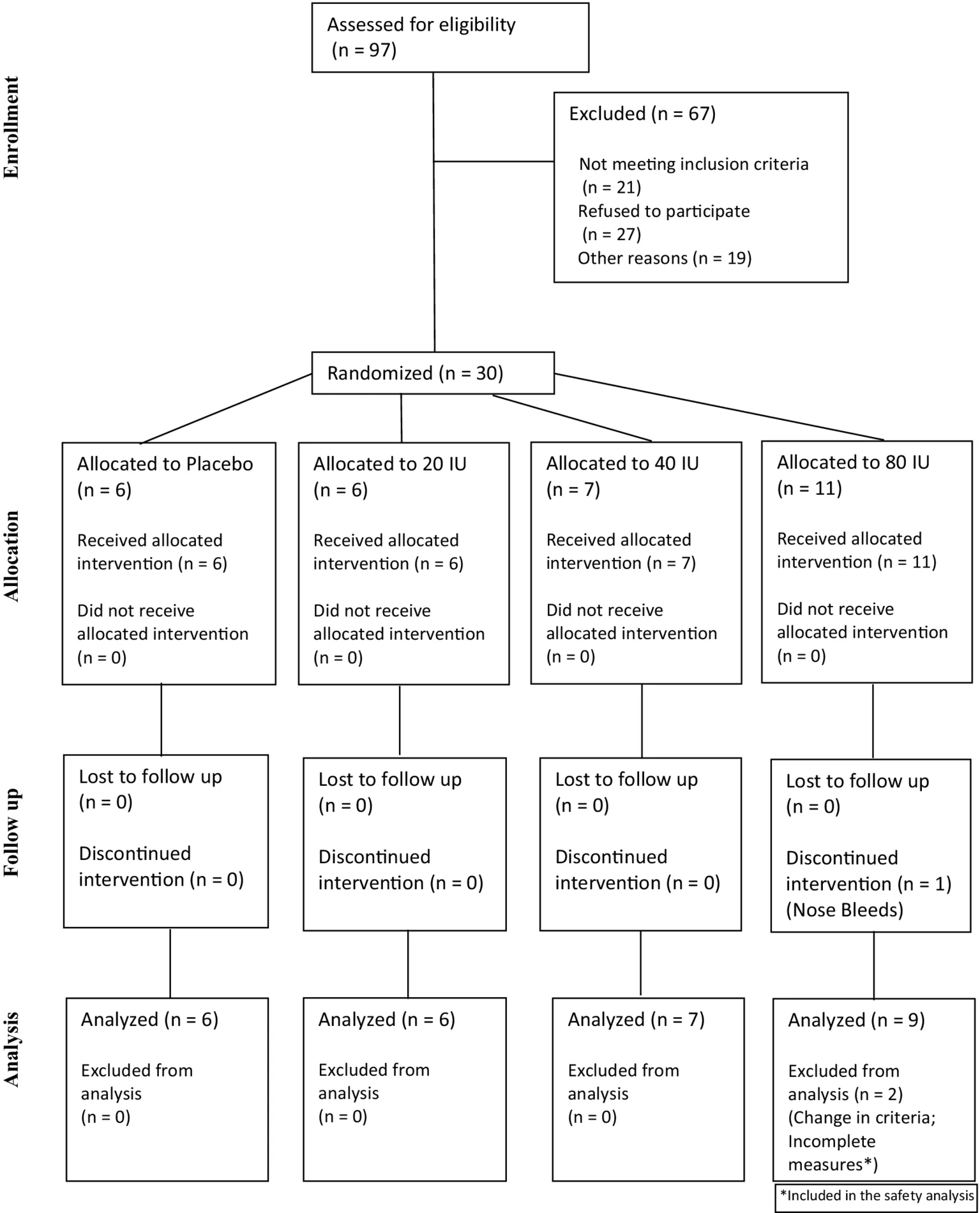
Study CONSORT diagram.

A total of 97 participants were contacted regarding study participation; of these, 31 were enrolled, 30 initiated treatments, and 29 completed treatments. One participant who signed the consent was deemed ineligible prior to randomization and did not initiate treatment. A total of 28 participants were included in the primary analytic cohort; one participant who completed treatment was removed following the revision of eligibility criteria around Montreal cognitive Assessment (MoCA (38)) score but is included in the safety analysis, as is the participant who did not complete treatment (Figure 2, consort). The eligibility criteria for the MoCA score were revised from ≥10 to ≥16 after completion of the first study participant. This modification was made in consultation with a neuropsychologist for two primary reasons, one, to minimize potential ceiling effects in cognitive testing, and second, to address the difficulty participants with MoCA scores below 16 experiences in completing the study’s cognitive assessments.

Participants were identified through a combination of targeted recruitment during scheduled clinic visits, internal promotion through organizational webpages, and newsletters distributed to Parkinson’s patients. Written informed consent was obtained from participants and assessed using study eligibility criteria. Participants were scheduled for a baseline visit if they were eligible at the screening visit.

Study participants had a diagnosis of idiopathic Parkinson’s Disease, confirmed by a movement disorders specialist, following MDS 2015 clinical diagnostic criteria (Hoehn and Yahr stage of </=3), were between 40 and 90 years of age (inclusive), and had a MoCA score of >/= 16 at screening. All study participants had a prior magnetic resonance imaging (MRI; obtained clinically prior to study participation) and agreed to remain stable on anti-parkinsonian medications for 30 days prior to randomization and throughout the treatment period. Participants with a history of significant head trauma, oto-pharyngeal surgery, chronic sinusitis, or severe deviated symptoms, insulin allergy were excluded from the trial.

### Study intervention

Insulin (Novolin-R, Novo Nordisk Inc) and placebo (0.9% bacteriostatic sodium chloride, 200 μL, each nostril, twice daily) were delivered using the SipNose intranasal delivery device (SipNose, Ltd., Israel (39)) for 21 days. Three different doses of insulin were used, with the highest number of participants (*n* = 9) in the group with the highest dose (80 IU/INI), a model previously used by Finger et al. (40). Dosing for the first two groups of this study is based on previously conducted INI studies in neurodegenerative disorders (26, 27), using daily doses of 20 and 40 IU of INI. Higher doses have been found to be safe in healthy adults (41). Prior studies performed have demonstrated favorable effects of this regimen in the MCI/AD population without peripheral hypoglycemia (26). Use of placebo control allows for clearer interpretation of the safety and tolerability of INI, given the studied population may have some baseline level of symptoms and/or deterioration in condition that could otherwise confound the results.

Participants in the active treatment arms received one of three different doses of insulin: 20 IU/INI (1O IU [100 μL]), one nostril, twice daily); 40 IU/INI (10 IU [100 μL], each nostril, twice daily); or 80 IU/INI (20 IU ([200 μL], each nostril, twice daily). The principal investigator, coordinator, and study staff who assessed outcomes as well as participants were blinded to the treatment assignment. Two members of the study team were unblinded and were responsible for preparing, dispensing, and collecting the study drug, as well as addressing any questions regarding the device or study drug, to maintain blinding for the rest of the study personnel. The first dose was completed in-person with unblinded staff to ensure correct use of the device without compromising blinding of the remaining study team. Allocation concealment was maintained using a centralized randomization scheme, with treatment assignments accessible only to designated unblinded study staff. To minimize the potential confounding effects of gender and MoCA score on the treatment effect, we used a 2 × 2 stratified randomization scheme with a block size of 4 when assigning patients to one of the four study groups. The stratification factors were gender (male/female) and MOCA score (low <22/30 and high>23/30). However, use of multiple stratification factors can (and did) lead to unequal treatment group sizes, especially with multiple treatment arms and small strata. Six participants were randomized to both placebo and 20 IU/INI arms, 7 to the 40 IU/INI arm, and 11 to the 80 IU/INI arm.

### Outcomes

Key outcomes included a composite safety event of either a reduction of fasting glucose to <70 mg/dL or an unintended reduction of weight >5% between any of screening, baseline or post treatment visits; any significant pre-post change in fasting glucose or weight; and adverse events (AEs) and serious adverse events (SAEs). Secondary outcomes included pre-post changes in cognition assessed with a neurocognitive battery (see Supplementary Table S1) (38, 42–49); motor symptoms measured with the Movement Disorder Society-Unified Parkinson’s Disease Rating Scale III [MDS-UPDRS; (50)]; and mood measured by the Beck Depression Inventory- Second Edition [BDI; (51)] and apathy as measured by the Apathy Scale (52).

### Statistical analysis

The repeated measures ANOVA (RM-ANOVA) model was used to test changes in body weight, fasting blood glucose, and insulin levels over time by treatment arm for significance; post-hoc pairwise comparisons were made using a Bonferroni correction. General linear mixed models with random person-specific intercepts were used to test for within-arm pre-post differences in any of the cognitive measures, mood, apathy and motor performance. In addition, a difference-in-differences (DiD) analysis framework using estimates from the Generalized Linear Mixed Model (GLMM) was used to test whether the active treatment arms improved significantly more than the placebo arm; this was accomplished by subtracting the pre-post placebo effect (meant to represent the natural within-person variation in responses associated with time and/or learning effects) from the pre-post treatment effects associated with each active arm. Appropriate to the exploratory nature of the secondary outcomes, we employed the False Discovery Rate (Benjamini-Hochberg) correction to control the proportion of false positives, while retaining high power to uncover potential relationships. All within-arm pre-post (4) cognitive assessments (19) were treated as one set of tests (4 × 19 = 76), and all DiD (3) cognitive assessments (19) were treated as another set (3 × 19 = 57); the BH correction was applied separately to each set. The procedure was repeated for non-cognitive tests (e.g., mood, apathy, and motor performance).

### Randomization

Stratified block randomization with a block size of 4 was used to assign participants to one of the 4 groups. The stratification factors were gender (male/female) and MoCA score (low <22/30 vs. high>23/30). The target sample size was 7 for each of the placebo, 20 IU/INI, and 40 IU/INI arms, and 9 for the 80 IU/INI arm.

## Results

### Study participants

Twenty-nine participants who met all the revised inclusion criteria (MoCA ≥ 16) were randomized, and 28 completed treatments. One participant did not complete treatment because of persistent nosebleeds; they are included in the safety outcomes analysis, but not the efficacy outcomes analysis. The average age of the 28 participants was 67.3 (SD = 5.5), with the average group age ranging from 63.4 (5.1) to 69.2 (6.1). Most participants were white (100%), non-Hispanic (96.4%) males (57.1%). The average MoCA score of all participants was 25.7 (2.7), and the average score in the placebo and 80 IU/INI arms was consistent with the mild cognitive impairment cutoff of <26; however, despite stratified randomization by MoCA, arms are somewhat imbalanced on MoCA score, complicating the interpretation of any cognitive improvements observed. MDS-UPDRS III and HbA1c were similarly distributed across groups, while average LEDD, a surrogate for PD severity, varied more widely (Table 1).

**Table 1:** Demographics by treatment group and overall.

Study Arm	Placebo	20 IU/INI	40 IU/INI	80 IU/INI	Overall
N	6	6	7	9	28
Age	69.2 (6.1)	68.3 (5.0)	63.4 (5.1)	68.2 (4.9)	67.3 (5.5)
Sex: Male	66.7% (*n* = 4)	50% (*n* = 3)	57.1% (*n* = 4)	55.6% (*n* = 5)	57.1% (*n* = 16)
Race: White	100% (*n* = 6)	100% (*n* = 6)	100% (*n* = 7)	100% (*n* = 9)	100% (*n* = 28)
Ethnicity: Not Hispanic	100% (*n* = 6)	100% (*n* = 6)	85.7% (*n* = 6)	100% (*n* = 9)	96.4% (*n* = 27)
MoCA	24.5 (1.9)	26.8 (1.8)	27.3 (2.7)	24.6 (3.0)	25.7 (2.7)
MDS-UPDRS III	24.3 (8.2)	24.2 (13.8)	22.9 (11.1)	24.4 (12.0)	24.0 (10.9)
LEDD	504 (193)	613 (215)	457 (318)	736 (373)	590 (304)
Baseline HbA1c	5.6 (0.2)	5.7 (0.2)	5.5 (0.2)	5.6 (0.5)	5.6 (0.3)

Continuous measures reported as Mean (SD), categorical as % (N).

N, Number of participants; MoCA, Montreal Cognitive Assessment, MDS-UPDRS III, Movement Disorders Society–Unified Parkinson’s Disease Rating Scale; LEDD, Levodopa Equivalent Daily Dose in mg; HbA1c, (Hemoglobin A1c).

### Safety outcomes

Overall, intranasal insulin was relatively safe and well tolerated in PD.

#### Change in weight

No participant experienced unintended weight loss of >5% of weight measured at screening. When analyzing body weight across the 3 timepoints, there were no main effects of treatment arm, time, nor interaction of the two. Post-hoc pairwise testing with Bonferroni adjustment showed that the 20 IU/INI group had a significantly lower body weight across all time points compared to all other study arms (*p* = 0.01); this was unrelated to the study drug and is instead a result of natural imbalance between the arms (Supplementary Table S2).

#### Change in fasting blood glucose level

Fasting blood glucose level was measured at baseline and post-treatment visits (Supplementary Table S3). There was a statistically significant decline in fasting glucose over time for all groups (i.e., a main effect for time, *p* = 0.004). The average decline across all groups was 3.5 mg/dL, a clinically insignificant level. There was not a significant difference between groups in terms of glucose level, nor was there a significant interaction between groups and time such that patterns differed between groups.

#### Change in point of care glucose (pre-post dose) level

No blood glucose values of <70 mg/dL were observed at pre-dose, 15-min post-dose and 30 min post-dose (Supplementary Table S4). There was a statistically significant decline in glucose levels over time for all groups (i.e., a main effect for time, *p* = 0.0006). There was not a significant difference between groups in terms of glucose level, nor was there a significant interaction between groups and time such that patterns differed between groups.

#### Change in insulin level

Insulin levels were measured at visits 2 and 3. Baseline insulin levels were within physiological range, though imbalance was observed (Supplementary Table S5). Given that within-person change in insulin was evaluated rather than improvement against a threshold, this imbalance was not concerning, particularly in the context of a randomized trial. There were no main effects of treatment arm, time, nor interaction of the two, meaning that arms did not differ at either visit 2 or visit 3; nor did insulin level change from visit 2 to visit 3 across all arms (Supplementary Table S5).

### Adverse events

There were 112 adverse events (AEs), 0 serious adverse events (SAEs), and 10 protocol deviations. Every participant reported at least one adverse event, with an average of 3.7 AEs per person. The average number of adverse events per person was nearly identical in the placebo and 20 IU/INI arms, and highest in the 40 IU/INI arm (5.0 per person). The average number of AEs per person in the 80 IU/INI arm was 3.8. No statistical comparisons are made between groups with respect to adverse event rates.

All participants experienced at least one mild adverse event, while a minority experienced at least one moderate adverse event (9–17% across arms). Only 1 participant (placebo arm) experienced a severe adverse event. The percentage of patients experiencing an adverse event that was probably related to the study drug was lowest in the placebo arm (67%) and similar across active treatment arms (82–86%). The 40 and 80 IU/INI arms were likelier to experience adverse events that were probably not related to the study drug (64–71%) than were the placebo or 20 IU/INI arms (33%). Adverse event details are provided in Supplementary Table S6–S9.

#### Nasal adverse events

Virtually all participants experienced at least one nasal adverse event (nasal irritation or nosebleeds, 83–100% by arm). However, nosebleeds and total nasal adverse events were least frequent in the 20 IU/INI group (Table 2). Nasal irritation was comparable between the placebo (50%), 20 IU/INI (67%), and 80 IU/INI (55%) arms, and notably higher in the 40 IU/INI (86%) arm.

**Table 2:** Nasal adverse events.

Study Arm	Participants with AE, *n* (%)		
	Nasal irritation	Nosebleeds	Nasal irritation or nosebleeds
Placebo	3 (50)	6 (100)	6 (100)
20 IU/INI	4 (67)	3 (50)	5 (83)
40 IU/INI	6 (86)	6 (86)	7 (100)
80 IU/INI	6 (55)	7 (64)	10 (91)

INI: Intranasal Insulin; AE: Adverse Events.

### Secondary/exploratory outcomes

#### Cognitive outcomes

In general, participants did not significantly change from baseline in most cognitive measures (Supplementary Table S10) once *p*-values were adjusted to control the false discovery rate. The exceptions were the Wechsler Memory Scale (WMS) delayed recall scores (+4.0) and the Stroop Word reading scores (+9.9), both in the 40 IU/INI arm only. It should be noted that the placebo arm also significantly improved in the WMS delayed recall (+2.5), but the improvement was no longer significant after adjustment. Of interest, the performance of the placebo group significantly declined from baseline on both the immediate and delayed recall portions of the Brief Visuospatial Memory Test (BVMT). Data quality assurance checks did not provide an explanation, and no placebo arm changes were significant after adjustment. Similarly, no difference-in-difference estimates (pre-post change of active arm less pre-post change of placebo arm) were significant after *p*-value adjustment (Supplementary Table S11), meaning that the two improvements in the 40 IU/INI arm described above were not significantly greater than that observed in the placebo arm.

#### Motor functioning

There were no significant changes over time in any of the treatment arms in motor function (Supplementary Table S12). One participant’s MDS-UPDRS-III score was incomplete at baseline; two sensitivity analyses that imputed the minimum possible score (13) and the maximum possible score (17) did not change the significance nor the interpretation of the treatment arm effects. All but two participants were on levodopa, and all motor exams were performed in the ON state.

#### Mood and apathy

There were no significant changes over time in any of the treatment arms in measures of depression and apathy (Supplementary Table S12).

## Discussion

Substantial safety data exist for intranasal insulin in humans; however, optimal dosing in patients with Parkinson’s disease was not evaluated previously. In this phase II trial, we evaluated the safety and tolerability of INI across three doses (20 IU/INI, 40 IU/INI, and 80 IU/INI) over a 21-day treatment period. Our findings indicate that INI is safe and well-tolerated following 21-days of treatment at doses of 20 IU/INI, 40 IU/INI and 80 IU/INI. It represents the largest INI trial conducted in PD to date that tested multiple doses with a robust safety profile, and it confirmed that participants could reliably self-administer INI.

The most comprehensive safety review to date was conducted by Schmid et al. (2018), who reviewed data from 1,092 participants receiving acute INI and 832 participants receiving chronic INI, with doses ranging from 10 IU/INI to 160 IU/INI (31). The nasal side effects observed in our study (e.g., mild irritation, epistaxis) were consistent with Schmid et al.’s findings (31). No clear dose–response relationship was detected for nasal adverse events, although event frequency was lowest at 20 IU/INI and highest at 40 IU/INI. This pattern may reflect the four-time-daily dosing regimen used in the placebo, 40 IU/INI, and 80 IU/INI groups. Notably, one participant with a history of nasal pathology withdrew early due to recurrent minor epistaxis, underscoring the importance of screening for pre-existing nasal conditions in future studies. Consistent with prior literature, including a 40 IU/INI PD trial by Novak et al. in 2019 (33), no hypoglycemic episodes, clinically significant serum glucose changes, or serious adverse events were observed. These data support the safety of INI at doses up to 80 IU/INI in PD.

Cognitive impairment is common in PD, often involving deficits in executive function, working memory, processing speed, visuospatial skills, and attention (1, 53). A key strength of the study included a comprehensive battery of neuropsychological tests that assessed cognition in PD and included targeted domains that have been shown to benefit from INI. However, improvement was observed in just two tests in the 40 IU/INI arm only, and not relative to placebo. Notably, verbal fluency (a measure of executive function) improved in the 40 IU/INI arm, consistent with the Novak et al. findings, although effects did not reach significance (33). Our ability to accurately assess the efficacy of INI on cognitive outcomes may have been complicated by variability in the timing of post-treatment assessments, some conducted more than 1 day after the last dose, which may have attenuated the observed cognitive effects (Supplementary Table S13). Unlike the Novak et al. study, we observed no significant changes in motor outcomes. No treatment effects were found for mood or apathy. Given that this trial was not powered to detect changes in outcomes (cognition, mood, or apathy), cognitive test results should be considered preliminary and hypothesis-generating, and future research should apply a stricter criterion (e.g., familywise error rate) in a targeted battery of cognitive outcomes to confirm estimates from this study. Furthermore, the imbalance in treatment arms at baseline on MoCA scores and LEDD values further complicates the interpretation of any observed increase in cognitive scores and underlines the importance of balance on these potential confounders and the need for future efficacy trials of INI in PD to be powered to detect treatment heterogeneity effects in certain subpopulations of interest.

Limitations include the relatively small sample size, multiple treatment arms, and a multiplicity of cognitive outcomes, all of which reduced statistical power in efficacy outcomes. If low enrollment remains prohibitive, future work may instead focus on comparing one or two active arms against a placebo control to first demonstrate efficacy of the treatment prior to dose-finding. This single-center study was almost completely non-Hispanic white participants, limiting the generalizability of its conclusions; a multi-center trial would increase generalizability and improve statistical power to detect efficacy. Another limitation is the use of short-acting insulin. While the half-life of intranasally administered Novolin-R (regular insulin) is not well established, INI has been detected in cerebrospinal fluid (21, 23) and clinical effects have been noted in the Parkinson’s population with regular intranasal insulin (31, 33). Due to the expected short half-life (54), we performed the post-treatment cognitive assessments within 1 day of administering the final dose in close to 80% of the participants (Supplementary Table S13). Still, the 21-day treatment period may have been insufficient to achieve sustained cognitive benefit, as noted in longer-duration trials in Alzheimer’s disease (28, 55). These findings indicate that longer duration (~6–12 months) of INI treatment may be necessary to adequately evaluate its effects on cognition. All participants on Levodopa were examined in the ON state, which may have reduced the ability to detect subtle treatment-related motor changes. Intranasal delivery devices use different administration strategies, which may have contributed to variations in outcomes compared with other studies utilizing INI (28, 56). Additionally, placebo effects were common, particularly for cognitive measures, a phenomenon well-documented in neurodegenerative disease trials, and potentially related to practice effects despite the use of alternate test forms (57). Other clinical trials, including some performed in Alzheimer’s disease, have described potential mechanisms for placebo response such as Rater’s expectations about scoring, or changes by improved care within a trial (58–60). Future studies should incorporate strategies to mitigate placebo responses.

## Conclusion

This phase II multi-dose study demonstrates that INI at daily doses of 20–80 IU/INI is safe, well tolerated, and associated with minimal nasal side effects in PD. Preliminary cognitive findings suggest possible benefits in select domains, supporting the rationale for larger, longer-duration clinical trials to determine whether INI can meaningfully impact cognition in PD. For a future trial it would be interesting to add biomarker-based assessments including neuroimaging, inflammatory markers, or insulin signaling biomarkers.

## Plain language summary

Parkinson’s disease (PD) is a brain disorder that causes movement problems and often affects thinking and memory. In this study, we tested whether a nasal (nose) spray of insulin, a hormone used to control blood sugar levels in people with diabetes, is safe for people with PD when delivered to the brain through the nose. Thirty people with PD were randomly assigned to receive either a placebo (saline nasal spray) or one of the three insulin doses (20, 40, 80 units) per day for 21 days. Our results showed that insulin nasal spray was safe at all doses tested, with no one having any low blood sugar events, or major changes in weight or insulin levels. Common side effects were nasal irritation and occasional mild nosebleeds. No serious side effects were reported. Participants also completed thinking and memory tests. Most test scores did not improve after insulin treatment. The 40-unit group showed improvements in two memory and thinking tests, but it is not clear whether these results are meaningful due to the small number of people in the study. There were no clear improvements in mood or movement symptoms. Overall, these suggest that insulin nasal spray is safe, and further research is needed to confirm whether insulin nasal spray can improve thinking and daily functioning in PD.

## Data Availability

The datasets presented in this article are not readily available because institutional policies do not allow providing data without execution of data agreement. Deidentified data is available from the corresponding author upon request and execution of relevant data agreement. Requests to access the datasets should be directed to bhavani.kashyap@healthpartners.com.
