# Supplementary material for: Safety and tolerability of intranasal insulin in Parkinson’s disease
Source: Front Neurol. 2026 Sep 9;17:1875165. doi: 10.3389/fneur.2026.1875165 (PMC13599274; doi:10.3389/fneur.2026.1875165)
Supplement: Supplementary file 1 [file Data_Sheet_1.pdf]

Supplemental Tables & Figures

S1: List of Neurocognitive Tests Administered at Baseline and Post-Treatment Visits

Category	Neurocognitive Assessment
**Global Cognition**	Montreal Cognitive Assessment (MoCA) [1, 2]
**Working Memory**	WAIS-IV Digit Span [3]
**Processing speed and Set-shifting**	Trailmaking Test [TMT] Part A and B [4]
**Visuospatial ability**	Judgement of Line Orientation [5]
**Learning and Memory**	Logical memory - Wechsler Memory Scale – Fourth Edition [6]
	Hopkins Verbal Learning Test- Revised (HVLT) [7]
	Brief Visuospatial Memory Test – Revised (BVMT) [8]
**Inhibition**	Stroop Color and Word Test [9]
	Letter Fluency [4]
	Category Fluency

S2: Weight and Weight Change While On-Study

Visit<br>Mean (SD)	Placebo	20 IU/INI	40 IU/INI	80 IU/INI
**Screening (lbs)**	180 (52.3)	167 (24.7)	179 (41.5)	194 (42.3)
**Baseline**	180 (51.8)	165 (23.9)	178 (41.4)	195 (42.2)
**% change from screening wgt**	-0.4% (0.8%)	-1.2% (0.9%)	-0.5% (1.5%)	0.7% (3.9%)
**Post-Treatment**	179 (50.3)	164 (24.3)	178 (42.5)	192 (43.6)
**% change from screening wgt**	-0.5% (1.5%)	-1.6% (0.8%)	-0.5% (1.1%)	0.8% (4.4%)

S3: Fasting Glucose Levels at Baseline and Post-Treatment Visits

Visits<br>Mean (SD)	Placebo	20 IU/INI	40 IU/INI	80 IU/INI
**Baseline**	96.3 (9.0)	93.2 (3.9)	95.3 (8.2)	98.5 (13.4)
**Post-Treatment**	92.5 (10.4)	89.3 (3.8)	94.0 (7.5)	94.1 (11.9)

S4: Pre- and Post-dose Glucose Levels

Time<br>Mean (SD)	Placebo	20 IU/INI	40 IU/INI	80 IU/INI
**Pre-dose**	96.8 (12.9)	116.0 (27.0)	102.0 (12.2)	114.0 (28.8)
**15 minutes**	94.7 (13.0)	112.0 (25.6)	93.9 (7.8)	108.0 (30.9)
**30 minutes**	94.7 (12.7)	106.0 (25.8)	90.7 (4.6)	105.0 (27.2)

S5: Fasting Insulin Levels at Baseline and Post-Treatment Visits

Visits<br>Mean (SD)	Placebo	20 IU/INI	40 IU/INI	80 IU/INI
**Baseline**	10.3 (9.6)	4.6 (0.9)	5.7 (3.1)	7.9 (6.0)
**Post-Treatment**	8.3 (7.8)	4.8 (1.7)	5.6 (4.0)	7.7 (6.3)

S6: Total Adverse Events - Group

Group	Average adverse AEs per person (SD)	Participants with at least 1 AE, n (%)
**Placebo**	3.0 (0.9)	6 (100)
**20 IU/INI**	2.8 (1.2)	6 (100)
**40 IU/INI**	5.0 (2.8)	7 (100)
**80 IU/INI[1]**	3.8 (2.0)	11 (100)
[1]n=11 participants in safety population for 80 IU/IN		

S7: Adverse Events – Participants with AE by Severity

	Participants with at least 1 AE, n (%)		
**Group**	**Mild**	**Moderate**	**Severe**
**Placebo**	6 (100)	1 (17)	1 (17)
**20 IU/INI**	6 (100)	1 (17)	0 (0)
**40 IU/INI**	7 (100)	1 (14)	0 (0)
**80 IU/INI [1]**	11 (100)	1 (9.1)	0 (0)
[1]n=11 participants in safety population for 80 IU/IN			

S8: Adverse Events – Participants with AE by Relationship to Study Drug

	Participants with at least 1 AE, n (%)			
**Group**	**Not related**	**Probably not related**	**Possibly related**	**Probably related**
**Placebo**	2 (33%)	2 (33%)	3 (50%)	4 (67%)
**20 IU/INI**	2 (33%)	2 (33%)	1 (33%)	5 (83%)
**40 IU/INI**	3 (43%)	5 (71%)	4 (57%)	6 (86%)
**80 IU/INI [1]**	3 (27%)	7 (64%)	4 (36%)	9 (82%)
[1]n=11 participants in safety population for 80 IU/IN				

S9: Adverse Events – Participants with AE by Body Systems

Body Systems, N (%)	Placebo	20 IU/INI	40 IU/INI	80 IU/INI[1]	Overall
**Nose bleeds**	6 (100)	3 (50)	6 (86)	7 (64)	22 (73)
**Nasal irritation**	6 (100)	4 (67)	6 (86)	6 (55)	19 (63)
**Other**	2 (33)	0 (0)	5 (71)	6 (55)	13 (43)
**Respiratory/sinus**	1 (17)	2 (33)	0 (0)	5 (45)	8 (27)
**Gastrointestinal issues**	1 (17)	0 (0)	1 (14)	2 (18)	4 (13)
**Headache**	0 (0)	0 (0)	2 (29)	2 (18)	4 (13)
**Falls**	1 (17)	1 (17)	0 (0)	0 (0)	2 (6.7)
**Increased Blood Pressure**	0 (0)	2 (33)	1 (14)	0 (0)	3 (10)
**Muscle aches, pains**	1 (17)	1 (17)	1 (14)	0 (0)	3 (10)
**Light-headed**	0 (0)	0 (0)	0 (0)	1 (9.1)	1 (3.3)

[1]n=11 participants in safety population for 80 IU/IN

S10: Cognitive Outcome Measures, Pre-Post Difference Within Treatment Arm

	Placebo			20 IU/INI			40 IU/INI			80 IU/INI		
**Cognitive Test**	**Pre**	**Post**	**Post-Pre**	**Pre**	**Post**	**Post-Pre**	**Pre**	**Post**	**Post-Pre**	**Pre**	**Post**	**Post-Pre**
**MoCA**	24.5	25.0	0.5 (-1.5, 2.5)	26.8	25.8	-1.0 (-3.0, 1.0)	27.3	26.6	-0.7 (-2.5, 1.1)	24.6	25.2	0.7 (-0.9, 2.3)
**JOLO[a]**	0.12	0.33	0.21 (-0.41, 0.84)	-0.08	-0.05	0.03 (-0.64, 0.70)	0.54	0.73	0.19 (-0.38, 0.77)	0.34	-0.09	-0.44 (-0.94, 0.07)
**TMT-A Time[b]**	47.0	52.8	5.8 (-1.9, 13.6)	46.7	51.2	4.5 (-3.3, 12.3)	45.3	50.6	5.3 (-1.9, 12.5)	46.6	51.3	4.8 (-1.6, 11.1)
**TMT-B Time[b]**	51.5	48.7	-2.8 (-9.3, 3.7)	42.2	45.7	3.5 (-3.0, 10.0)	51.7	57.3	5.6 (-0.4, 11.6)	49.0	51.1	2.1 (-3.2, 7.4)
**WMS Immediate Recall[c]**	8.5	12.0	3.5* (1.1, 5.9)	10.2	12.8	2.7* (0.2, 5.1)	10.3	12.7	2.4* (0.2, 4.7)	10.6	11.9	1.3 (-0.7, 3.3)
**WMS Delayed Recall[c]**	9.3	11.8	2.5* (0.7, 4.3)	10.0	11.7	1.7 (-0.2, 3.5)	9.7	13.7	4.0**, ^^ (2.3, 5.7)	9.8	12.2	2.4* (1.0, 3.9)
**WMS Recognition[d,‡]**	26-50 (5)	>75 (6.5)	1.5	51-75 (6)	>75 (7)	1	>75 (7)	>75 (7)	0	51-75 (6)	51-75 (6)	0
**COWAT Total[b]**	49.5	50.8	1.3 (-4.6, 7.3)	46.2	50.8	4.7 (-1.3, 10.6)	47.0	52.0	5.0 (-0.5, 10.5)	50.9	53.8	2.9 (-2.0, 7.8)
**COWAT Animals[b]**	52.3	53.8	1.5 (-5.1, 8.1)	39.8	46.5	6.7 (0.12, 13.2)	49.1	53.9	4.7 (-1.4, 10.8)	53.8	52.0	-1.8 (-7.1, 3.6)
**BVMT Immediate Recall[b]**	54.5	42.7	-11.8** (-19.6, -4.1)	47.3	52.8	5.5 (-2.2, 13.2)	53.4	44.9	-8.6* (-15.7, -1.4)	46.9	43.9	-3.0 (-9.3, 3.3)
**BVMT Delayed Recall[b]**	54.8	45.2	-9.7** (-17.7, -2.2)	52.5	49.7	-2.8 (-10.3, 4.7)	55.6	53.9	-1.7 (-8.7, 5.2)	49.7	47.3	-2.3 (-8.5, 3.8)
**BVMT Recognition[e‡]**	>16 (6)	>16 (6)	0	>16 (6)	>16 (6)	0	>16 (6)	>16 (6)	0	>16 (6)	11-16 (6)	-1
**HVLT Immediate Recall[b]**	48.0	48.0	0.0 (-8.0, 8.0)	44.5	47.5	3.0 (-5.0, 11.0)	46.3	47.9	1.6 (-5.8, 9.0)	47.1	42.6	-4.6 (-11.1, 2.0)
**HVLT Delayed Recall[b]**	49.8	50.3	0.5 (-7.8, 8.8)	46.5	46.3	-0.2 (-8.5, 8.2)	47.3	50.4	3.1 (-4.6, 10.8)	47.7	42.4	-5.2 (-12.0, 1.6)
**HVLT Recognition[b]**	44.0	45.3	1.3 (-8.5, 11.2)	48.3	50.5	2.2 (-7.7, 12.0)	50.7	46.3	-4.4 (-13.5, 4.7)	46.0	38.8	-7.2 (-15.3, 0.8)
**Stroop Word Reading[b]**	35.2	36.8	1.7 (-2.9, 6.3)	36.3	35.5	-0.8 (-5.4, 3.8)	34.9	44.7	9.9**, ^^ (5.6, 14.1)	39.2	42.0	2.8 (-0.1, 6.5)
**Stroop Color Reading[b]**	40.7	43.2	2.5 (-1.9, 6.9)	40.0	37.7	-2.3 (-6.7, 2.0)	38.9	45.1	6.3** (2.2, 10.3)	38.8	40.6	1.8 (-1.8, 5.4)
**Stroop Color-Word[b]**	47.2	50.0	2.8	44.3	45.3	1.0	50.7	53.0	2.3	48.9	47.2	-1.7

			(-1.0, 6.7)			(-2.8, 4.8)			(-1.3, 5.8)			(-4.8, 1.5)
**Stroop Interference[b]**	49.7	51.0	1.3<br>(-2.7, 5.3)	46.0	48.2	2.2<br>(-1.8, 6.2)	55.6	52.7	-2.9<br>(-6.6, 0.9)	51.2	49.4	-1.8<br>(-5.1, 1.5)
**Forward Digit Span[c]**	11.2	12.2	1.0<br>(-0.8, 2.8)	10.2	11.7	1.5<br>(-0.3, 3.3)	9.7	10.7	1.0<br>(-0.7, 2.7)	10.2	10.4	0.2<br>(-1.3, 1.7)
**Backward Digit Span[c]**	10.5	12.8	2.3*<br>(0.3, 4.4)	12.3	12.3	0.0<br>(-2.0, 2.0)	10.6	10.1	-0.4<br>(-2.3, 1.5)	10.4	10.4	0.0<br>(-1.7, 1.7)

[a] Z-score; [b] T-score; [c] Scaled Score; [d] Scaled Score, categorized; [e] T-score, categorized; ‡ Median category shown (ordinal category in parentheses); Wilcoxon signed rank test for ordinal data used to test for pre/post improvement over time (all nonsignificant, 95% CIs not available); Model-based means, 95% CIs from general linear mixed models. Uncorrected *p<0.05, **p<0.01; after correction using Benjamini-Hochberg FDR, ^p<0.05, ^^p<0.01

S11: Change in Cognitive Outcomes in Insulin, Relative to Placebo (Difference-in-Differences)

	**20 IU/INI**		**40 IU/INI**		**80 IU/INI**	
**Cognitive Test**	DID estimate (20 IU/IN change – Placebo change)	DID *p* value	DID estimate (40 IU/IN change – Placebo change)	DID *p* value	DID estimate (80 IU/IN change – Placebo change)	DID *p* value
**MoCA**	-1.5<br>(-4.3, 1.3)	0.27	-1.2<br>(-3.9, 1.5)	0.36	0.<br>(-2.4, 2.7)	0.89
**JOLO[a]**	-0.18<br>(-1.1, 0.73)	0.68	-0.02<br>(-0.87, 0.83)	0.96	-0.65<br>(-1.5, 0.15)	0.11
**TMT-A Time[b]**	-1.3<br>(-12.3, 9.6)	0.80	-0.6<br>(-11.1, 10.0)	0.92	-1.1<br>(-11.1, 9.0)	0.83
**TMT-B Time[b]**	6.3<br>(-2.8, 15.5)	0.17	8.4<br>(-0.4, 17.2)	0.06	4.9<br>(-3.4, 13.3)	0.23
**WMS Immediate Recall[c]**	-0.8<br>(-4.3, 2.6)	0.62	-1.1<br>(-4.4, 2.3)	0.51	-2.2<br>(-5.3, 1.0)	0.17
**WMS Delayed Recall[c]**	-0.8<br>(-3.4, 1.7)	0.51	1.5<br>(-1.0, 4.0)	0.22	-0.1<br>(-2.4, 2.3)	0.96
**WMS Recognition[d]**	Categorical; cannot be modeled as contrast					
**COWAT Total[b]**	3.3<br>(-5.1, 11.8)	0.42	3.7<br>(-4.5, 11.8)	0.36	1.6<br>(-6.2, 9.3)	0.68
**COWAT Animals[b]**	5.2<br>(-4.1, 14.4)	0.26	3.2<br>(-5.7, 12.1)	0.46	-3.3<br>(-11.7, 5.2)	0.43
**BVMT Immediate Recall[b]**	17.3**<br>(6.4, 28.3)	<0.01	3.3<br>(-7.3, 13.8)	0.53	8.8<br>(-1.1, 18.8)	0.08
**BVMT Delayed Recall[b]**	6.8	0.20	8.0	0.12	7.3	0.13

	(-3.8, 17.5)		(-2.3, 18.2)		(-2.4, 17.0)	
**BVMT Recognition[e]**	Categorical; cannot be modeled as contrast					
**HVLT Immediate Recall[b]**	3.0<br>(-8.3, 14.3)	0.59	1.6<br>(-9.3, 12.5)	0.77	-4.6<br>(-14.9, 5.8)	0.37
**HVLT Delayed Recall[b]**	-0.7<br>(-12.4, 11.1)	0.91	2.6<br>(-8.7, 14.0)	0.63	-5.7<br>(-16.5, 5.0)	0.28
**HVLT Recognition[b]**	0.8<br>(-13.1, 14.8)	0.90	-5.8<br>(-19.2, 7.7)	0.38	-8.6<br>(-21.3, 4.2)	0.18
**Stroop Word Reading[b]**	-2.5<br>(-9.0, 4.0)	0.43	8.2*<br>(1.9, 14.5)	0.01	1.1<br>(-4.8, 7.0)	0.70
**Stroop Color Reading[b]**	-4.8<br>(-11.0, 1.4)	0.12	3.8<br>(-2.2, 9.8)	0.20	-0.7<br>(-6.4, 4.9)	0.79
**Stroop Color-Word[b]**	-1.8<br>(-7.2, 3.6)	0.49	-0.6<br>(-5.8, 4.7)	0.83	-4.5<br>(-9.4, 0.4)	0.07
**Stroop Interference[b]**	0.8<br>(-4.8, 6.5)	0.76	-4.2<br>(-9.7, 1.3)	0.13	-3.1<br>(-8.3, 2.1)	0.23
**Forward Digit Span[c]**	0.5<br>(-2.1, 3.1)	0.69	0.0<br>(-2.5, 2.5)	0.99	-0.8<br>(-3.1, 1.6)	0.50
**Backward Digit Span[c]**	-2.3<br>(-5.2, 0.6)	0.11	-2.8<br>(-5.6, 0)	0.05	-2.3<br>(-5.0, 0.3)	0.08
**Beck Depression Inventory**	-1.3<br>(-5.6, 2.9)	0.52	-1.1<br>(-5.2, 3.0)	0.58	1.4<br>(-2.5, 5.2)	0.46
**Apathy Scale**	-0.7<br>(-6.3, 4.9)	0.81	-2.2<br>(-7.6, 3.2)	0.41	0.3<br>(-4.8, 5.4)	0.89
**MDS-UPDRS-III[‡]**	0.3<br>(-11.3, 11.9)	0.95	2.5<br>(-8.7, 13.7)	0.65	-3.2<br>(-13.8, 7.4)	0.54

[a] Z-score; [b]T-score; [c] Scaled Score; [d] Scaled Score, categorized; [e] T-score, categorized; [‡] One participant's MDS-UPDRS-III score was incomplete at baseline; two sensitivity analyses that imputed the minimum possible score (13) and the maximum possible score (17) did not change the significance nor the interpretation of the treatment arm effects. Model-based means, 95% CIs from general linear mixed models. Uncorrected *p<0.05, **p<0.01; after correction using Benjamini-Hochberg FDR, ^p<0.05, ^^p<0.01

S12: Non-Cognitive Outcome Measures

	Placebo			20 IU/INI			40 IU/INI			80 IU/INI		
**Test**	**Pre**	**Post**	**Post-Pre**	**Pre**	**Post**	**Post-Pre**	**Pre**	**Post**	**Post-Pre**	**Pre**	**Post**	**Post-Pre**
**MDS-UPDRS-III[a]**	24.3	23.8	-0.5 (-8.7, 7.7)	24.2	24.0	-0.2 (-8.4, 8.0)	22.9	24.9	2.0 (-5.6, 9.6)	24.4	20.8	-3.7 (-10.4, 3.0)
**Beck Depression Inventory**	9.0	7.8	-1.2 (-4.2, 1.8)	8.2	5.7	-2.5 (-5.5, 0.5)	7.4	5.1	-2.3 (-5.1, 0.5)	6.9	7.1	0.2 (-2.2, 2.7)
**Apathy Scale**	13.7	15.0	1.3 (-2.6, 5.3)	12.2	12.8	0.7 (-3.3, 4.6)	11.6	10.7	-0.9 (-4.5, 2.8)	11.2	12.9	1.7 (-1.6, 4.9)

[a] One participant's MDS-UPDRS-III score was incomplete at baseline; two sensitivity analyses that imputed the minimum possible score (13) and the maximum possible score (17) did not change the significance nor the interpretation of the treatment arm effects. Model-based means, 95% CIs from general linear mixed models. *p<0.05, **p<0.01; after correction using Benjamini-Hochberg FDR, ^p<0.05, ^^p<0.01

S13: Days between Final Dose and Final Cognitive Assessment

Days between Final Dose and Final Cognitive Assessment	
**Mean ± SD**	1.7 (1.68)
**Median**	1
**% of Participants with 1 day**	80

References

1. Hoops S, Nazem S, Siderowf A, et al. Validity of the MoCA and MMSE in the detection of MCI and dementia in Parkinson disease. *Neurology* 2009; 73: 1738-1745.
2. Nasreddine ZS, Phillips NA, Bédirian V, et al. The Montreal Cognitive Assessment, MoCA: a brief screening tool for mild cognitive impairment. *Journal of the American Geriatrics Society* 2005; 53: 695-699.
3. Wechsler D. *Wechsler adult intelligence scale (WAIS-IV)*. Pearson, The Psychological Corporation, 2008.
4. Heaton RK. *Revised comprehensive norms for an expanded Halstead-Reitan Battery: Demographically adjusted neuropsychological norms for African American and Caucasian adults, professional manual*. Psychological Assessment Resources, 2004.
5. Benton AL, Varney NR and Hamsher Kd. Visuospatial judgment: A clinical test. *Archives of neurology* 1978; 35: 364-367.
6. Wechsler D. Wechsler memory scale–third edition (WMS-III). *San Antonio, TX: The Psychological Corporation* 1997.
7. Brandt J. The Hopkins Verbal Learning Test: Development of a new memory test with six equivalent forms. *The clinical neuropsychologist* 1991; 5: 125-142.
8. Benedict RH. *Brief visuospatial memory test--revised*. PAR, 1997.
9. Golden CJ and Freshwater SM. Stroop color and word test kit for adults. 2002.
